# Trace metal release after minimally-invasive repair of pectus excavatum

**DOI:** 10.1371/journal.pone.0186323

**Published:** 2017-10-12

**Authors:** Caroline Fortmann, Thomas Göen, Marcus Krüger, Benno M. Ure, Claus Petersen, Joachim F. Kübler

**Affiliations:** 1 Department of Pediatric Surgery, Hannover Medical School, Hannover, Germany; 2 Institute and Outpatient Clinic of Occupational, Social and Environmental Medicine, Friedrich-Alexander-University Erlangen-Nürnberg, Erlangen, Germany; 3 Department of Cardiothoracic, Transplantation and Vascular Surgery, Hannover Medical School, Hannover, Germany; Baylor College of Medicine, UNITED STATES

## Abstract

**Background:**

Several studies have shown a high incidence of metal allergy after minimally-invasive repair of pectus excavatum (MIRPE). We postulated that MIRPE is associated with a significant release of trace metal ions, possibly causing the allergic symptoms.

**Methods:**

We evaluated the concentration with chromium, cobalt and nickel in blood, urine and tissue in patients prior to MIRPE and in patients who underwent an explantation of the stainless-steel bar(s) after three years.

**Results:**

Our study group consisted of 20 patients (mean age 19 years) who had bar explantation and our control group included 20 patients (mean age 16 years) prior to MIRPE. At the time of bar removal we detected significantly elevated concentrations of chromium and nickel in the tissue compared to patients prior to the procedure (p<0,001). We also found a significant increase in the levels of chromium in urine and nickel in blood in patients three years post MIRPE (p<0,001). Four patients temporarily developed symptoms of metal allergy, all had elevated metal values in blood and urine at explantation.

**Conclusions:**

Minimally-invasive repair of pectus excavatum can lead to a significant trace metal exposure.

## Introduction

The minimally invasive repair of pectus excavatum (MIRPE) via the implantation of one or more metal bars has become the standard technique for correction of pectus excavatum morphology. Correction is performed primarily for cosmetic reasons and to ease any psychological strain that is associated with body image concerns [1–3]. To elevate the sternum one or more metal bars are placed substernally via small bilateral thoracic incisions under thoracoscopic guidance [4–6]. The bars subsequently remain in place for approximately three years. There are different methods of bar fixation using stabilizers, bridging metal plates, steel wire or suture.

Over the past ten years, several publications have reported a high incidence of local tissue reactions after MIRPE without clear signs of infection. These reactions occur in 6–8% of the cases and have been attributed to an allergic reaction to the metal bars implanted [7, 8]. However, some patients have developed these symptoms despite a negative preoperative skin test [7–9]. Treatment usually includes the administration of cortisone and antibiotics and, in most cases the symptoms decrease over time [7, 10].

Nonetheless, the causes of the high incidence of the local reactions that are obviously related to the implanted metal bars remain unclear. However, the potential of metal implants to cause local and systemic complications is well known in other medical application of metal prostheses. One prominent case involved the recall of the DePuy hip replacement, a case that attracted significant media coverage and resulted in massive medicolegal consequences for the companies involved [11, 12]. In these cases, it was determined that metallic debris had been released following implantation of a metal-on-metal hip replacement and that this debris caused severe tissue and bone damage, leading to an early failure of approximately 40% of the implants [12–14]. Many patients subsequently required revision surgery and some patients suffered from serious long-term complications [11]. Moreover, several studies found that the metal exposure was not restricted to the local tissue but showed significantly elevated levels of chromium and cobalt after metal-on-metal hip arthroplasty in blood and/or urine [15–18]. Accordingly, some of the patients developed symptoms of systemic metal toxicity such as cardiac or neurological symptoms.

As the pectus bar system used for MIRPE in our institution also has metal on metal surfaces, and because we and others observed a high incidence of nonspecific inflammatory responses, we hypothesized that implantation of stainless steel pectus bars can lead to local and systemic metal contamination as well.

## Material and methods

To evaluate potential metal contamination following the implantation of metal bars to repair pectus excavatum, we prospectively collected blood, urine, and tissue from patients before and after MIRPE. Two study groups were involved: a group of consecutive patients who underwent metal bar explantation three years after MIRPE, and a control group that consisted of patients prior to MIRPE between March 2015 and March 2016. The patients’ characteristics (age, sex, number and size of bars) were recorded prospectively. Development of allergic symptoms in the explantation group was collected retrospectively from the patient’s electronic chart. A stainless steel allergy test plate was applied preoperatively. The institutional review board of the Hannover Medical School approved this study (No. 6659), and all patients and legal guards gave their informed consent.

Metal bar implantation was performed via two lateral incisions and under thoracoscopic guidance. The bar (MedXpert, Germany), which consists of 1.4441 ASTM F 138 stainless steel [19], was first bent individually to the shape of the patient’s chest using a Pectus bar tabletop bender. Placement of the bar involved entering the thoracic cavity on the right side of the anterior chestwall, passing across the mediastinum, directly underneath the sternum and exiting the thorax on the left side. Finally, the middle part of the bar is placed substernally in contact with the pleura, and the lateral parts of the bar are fixated in the soft tissue using metal stabilizers. The stabilizers are secured for dislocation using a pin at the end of the bar, but still able to move along the bar. For removal of the bar, the lateral incisions were re-opened, the stabilizers removed and the bar was bent straight to be pulled out safely. There was no routinely follow-up in the explantation group, patients presented when developing symptoms. The main components of the stainless steel bar are chromium (18%) and nickel (15%), but many other metals and semimetals are also contained in very small amounts (e.g. 3% molybdenum, 2% manganese, 0.5% silicium, 0.2% cobalt, 0.2% copper and 0,01% aluminum).

The tissue sample (approx. ½ cm^3^), which consisted of subcutaneous tissue, was resected from the lateral chest wall in place where the stabilizer was fixated. In the control group this exact location was identified and the sample was taken before implantation of the metal. Urine (2 x 10 ml) was collected a few hours before the surgery and the blood sample (2 x 7,5 ml) was taken during induction of anesthesia right before the operation. Utilized collection tubes and cannulas were tested to be free of any trace metal contamination.

For monitoring of metal exposure the levels of chromium, cobalt and nickel were determined in all samples. For chromium monitoring the blood sample was separated into plasma and erythrocyte fractions. The chromium, cobalt and nickel content present in the patients’ tissue, urine and blood compartment was assessed using an inductively coupled plasma mass spectrometer (ICP-MS) with collision cell (Agilent 7500cx). Before ICP-MS analysis tissue samples underwent an acidic thermic digestion. For ICP-MS plasma generation, argon of 99.996% purity (AirLiquide) was used. A background reduction was performed for chromium and nickel using helium (99.999% pure, AirLiquide) as collision gas. Urine, blood and tissue digestion solution were prepared by diluting these materials 1:10 with a 0.2% nitric acid solution. Calibration standards were prepared in matrix blanks and spiked with incremental quantities of chromium, cobalt and nickel. Quantification of chromium, cobalt and nickel was performed by monitoring the ion mass 52 (chromium), 59 (cobalt) and 60 (nickel), respectively. For quality assurance purposes, quality control material (spiked matrix blanks) was analyzed in each series and assessed by quality control chart analysis. Analytical accuracy was assured by ensuring the procedures were conducted in accordance with the GEQUAS proficiency tests [20] and application to certified reference material (NIST 2668).

All data analyses were performed with SigmaStat^®^ by Jandel Scientific. Descriptive statistics were reported as mean ± standard deviation and range for continuous variables and as percentages for categorical variables. Statistical analyses in which the two groups were compared were then performed using the Mann-Whitney U test. A p-value of less than 0.05 was considered to be statistically significant. Due to the lack of studies there was no data for performing a power analysis. We did our first analysis after 20 patients, which already showed significant results.

Reference values for the tolerable level of metal in blood and urine in the general German population were employed to compare and classify the detected systemic metal values [21].

## Results

Our data included 20 consecutive patients who received a pectus bar explantation (study group) and 20 consecutive patients who underwent MIRPE (control group). Table 1 presents the demographic data. The mean age of the patients in the study group was 19.3 years (± 2.8 years; range 16–28 years) and 70% were male. The bar explantation was performed after a mean time of 37 months (± 4.4 month; range 29–48 months) and the majority of the patients (90%) had one bar in place. Two patients had two bars. Mean size of the bar(s) was 14 inches (± 1.6 inches; range 10–16 inches). All bars were fixed with two stabilizers, secured by a pin. The participants in the control group were significantly younger (p<0,001), the mean age was 16.3 years (± 6.0 years; range 11–41 years), and 90% were male (p = 0,123). All preoperative allergy tests were negative in both groups.

**Table 1:** Demographic data of all patients of the control and study group.

	Mean age (years)	Male: female
**Prior to MIRPE (control group) n = 20**	16.3	18: 2
**Bar removal (study group) n = 20**	19.2	14: 6
**P value**	<0,001	0,123

One patient was excluded from the consecutive control group because the exact time point of blood and tissue sample collection had not been documented.

### Contamination in tissue

**Fig 1:**
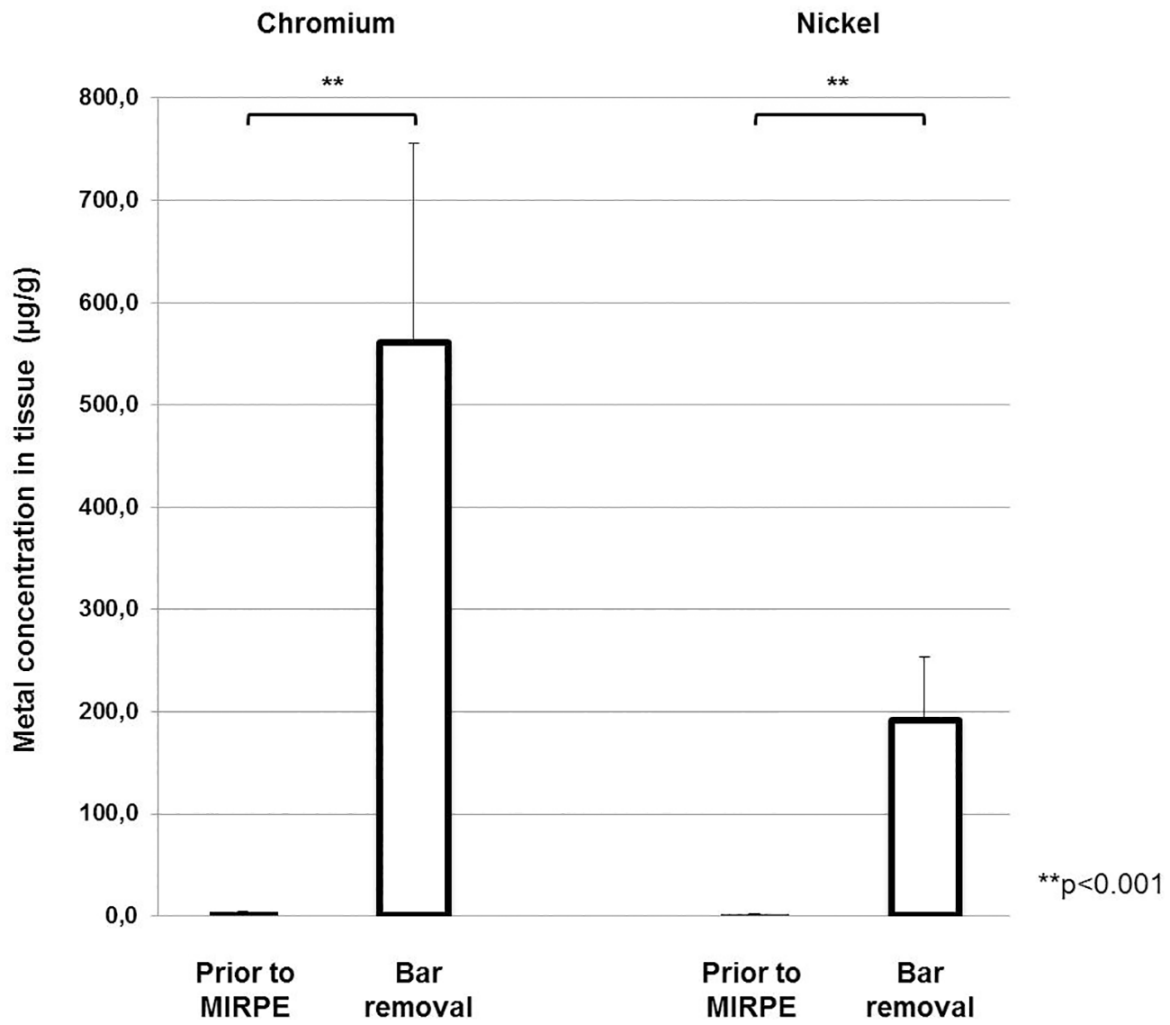
Chromium and nickel concentrations in tissue preoperatively and at the time of bar removal. Bars show mean concentrations of chromium and nickel in tissue and error bars show standard error of the mean. The differences between the concentrations preoperatively and at the time of bar removal were statistically significant (**p<0.001).

Highly elevated concentrations of chromium and nickel were found in the tissue surrounding the bar at the time of explantation in comparison to the metal levels in the tissue prior to MIRPE. Before metal bar implantation, the nickel and chromium concentration was 1.9 μg/g and 3.9 μg/g respectively. When the bar(s) had been in place for three years, these values increased to 190.7 μg/g and 560.7 μg/g respectively. These differences were statistically significant (p<0.001) and are demonstrated in Table 2 and Fig 1. The highest values that were measured at the time of bar removal were 2789 μg/g for chromium and 977 μg/g for nickel.

**Table 2:** Chromium and nickel contamination in tissue preoperatively and at the time of bar removal.

	Mean level prior to MIRPE (μg/g)	Mean level at bar removal (μg/g)	P value	Highest value at bar removal (μg/g)
**Chromium in tissue**	3.9	560.7	<0.001	2789
**Nickel in tissue**	1.9	190.7	<0.001	977

**Fig 2:**
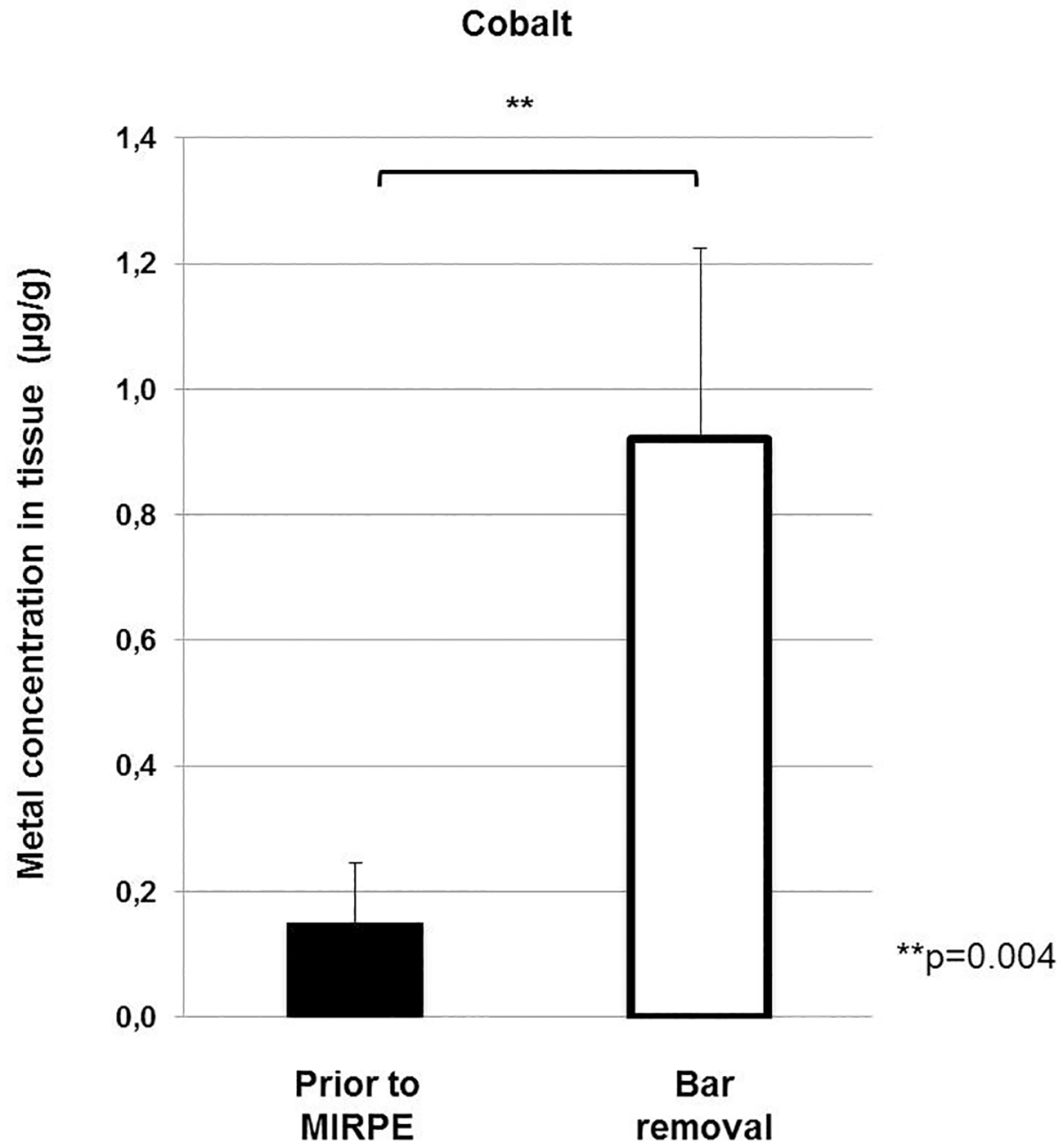
Cobalt concentrations in tissue preoperatively and at the time of bar removal. Bars show mean concentrations of cobalt in tissue and error bars show standard error of the mean. The difference between the concentrations preoperatively and at the time of bar removal was statistically significant (**p = 0.004).

We also observed a significant increase for the amount of cobalt in the tissue samples from 0.2 μg/g to 0.92 μg/g (p = 0.004), which is shown in Fig 2. Cobalt was just measured in the first 11 tissue samples of the study group and the first 9 patients of the control group and then discontinued due to absent elevated systemic values.

### Systemic exposure

**Fig 3:**
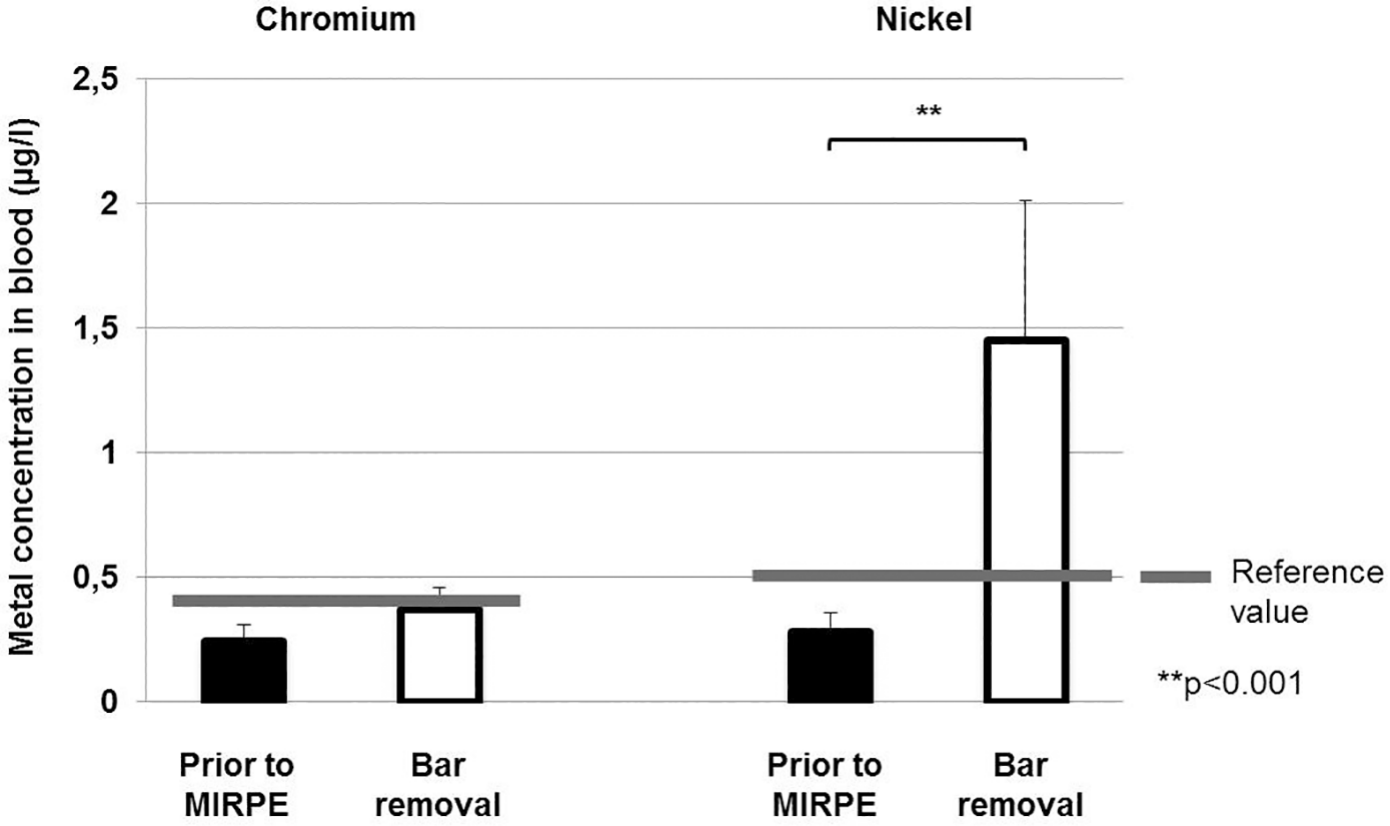
Chromium and nickel concentrations in blood preoperatively and at the time of bar removal. Bars show mean concentrations of chromium and nickel in blood and error bars show standard error of the mean. The difference between nickel concentrations preoperatively and at the time of bar removal was statistically significant (**p<0.001).

**Fig 4:**
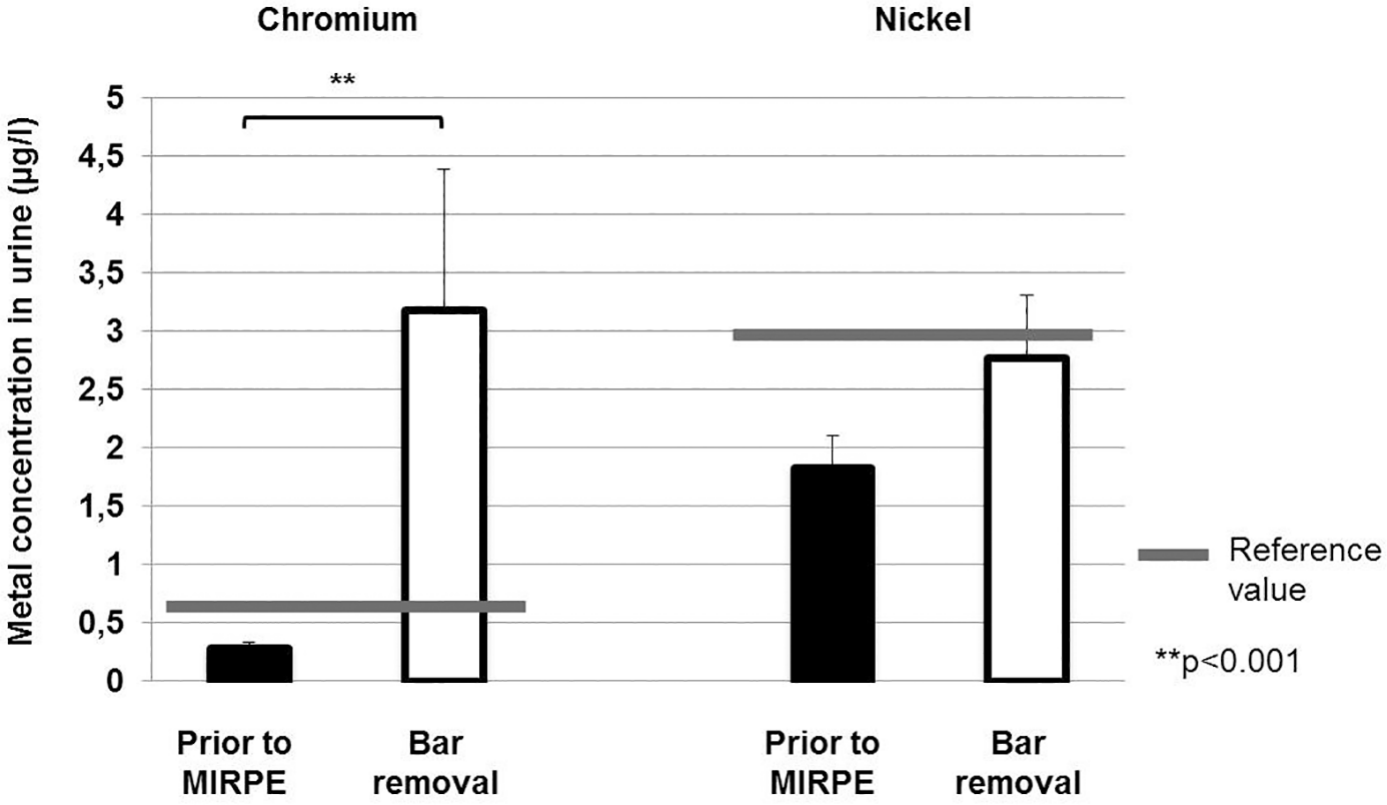
Chromium and nickel concentrations in urine preoperatively and at the time of bar removal. Bars show mean concentrations of chromium and nickel in urine and error bars show standard error of the mean. The difference between chromium concentrations preoperatively and at the time of bar removal was statistically significant (**p<0.001).

Also the systemic metal levels of chromium and nickel were elevated three years after implantation of the metal bar(s). The increase in the level of chromium in urine was especially striking, with patients exhibiting levels that were elevated by as much as 66 times that of the control values (highest value: 18.6 μg/l). The increase in the mean levels of chromium in urine was highly significant from 0.29 μg/l before implantation to 3.19 μg/l at the time of explantation (p>0.001), considerably higher than the reference value of 0.6 μg/l. For mean chromium values in plasma we found a slight increase from 0.24 μg/l before implantation to 0.37 μg/l at the time of explantation, but this difference was not statistically significant (p = 0.142). The highest value that was measured for chromium in plasma in the study group was 1.2 μg/l, significant above the reference value of 0.4 μg/l. In terms of nickel in blood, an increase was found from 0.28 μg/l pre implantation to 1.45 μg/l at explantation, with the highest value being 10.65 μg/l. This difference was statistically significant (p<0.001) and the levels at explantation were considerably higher than the reference value of 0.5 μg/l. Nickel levels in urine were just slightly elevated from 1.82 μg/l before bar implantation to 2.77 μg/l at bar removal, this difference was not statistically significant (p = 0.379). But the highest value for nickel in urine that was measured at explantation (8.9 μg/l) was much higher than the reference value of 3 μg/l. The systemic chromium and nickel levels in both groups are presented in Table 3 as well as Figs 3 and 4.

**Table 3:** Chromium and nickel concentration in blood and urine preoperatively and at the time of bar removal.

	Mean level prior to MIRPE (μg/l)	Mean level at bar removal (μg/l)	P value	Highest level at bar removal (μg/l)	Reference value (μg/l)
**Chromium in plasma**	0.24	0.37	0.142	1.2	0.4
**Chromium in urine**	0.29	3.19	<0.001	18.6	0.6
**Nickel in blood**	0.28	1.45	<0.001	10.65	0.5
**Nickel in urine**	1.82	2.77	0.379	8.9	3

We did not detect a significant increase for the cobalt concentrations in blood and urine samples at bar removal. The mean cobalt levels in blood were 0.15 μg/l before implantation and 0.21 μg/l at explantation (p = 1.0), the values in urine were 0.65 μg/l prior to MIRPE and 0.88 μg/l at bar removal (p = 0.371). All these concentrations in both groups were below the reference values (0,5 μg/l for cobalt in blood, and 1,5 μg/l in urine). Due to this finding we discontinued measuring cobalt levels after 16 patients of the control group and 14 patients of the study group.

### Symptoms

In the bar removal group 4 patients had symptoms during the postoperative course that could be related to metal allergy, no bar had to be explanted premature. The symptoms occurred after a mean time of 14 days (range 8 to 22 days) after bar implantation and consisted of recurrent pain and uni- or bilateral pleural effusion in all cases. All of these patients received antibiotics and two additional prednisolone. Skin reactions were not documented. At the time of bar removal all of these patients had elevated values for chromium and/ or nickel in blood and/ or urine, but due to the retrospective data collection of this patient group we don’t know the level of the nickel and chromium concentrations at the time of the symptoms.

## Discussion

Our observation indicates that MIRPE with placement of intrapleural and substernal metal bars can lead to a significant release of trace metal ions. We observed a significant local and systemic contamination with nickel and chromium.

The first report of an allergy after MIRPE was published in 2007 as a retrospective review. Rushing et al. found a 2.2% incidence of metal allergy over an 18-year period that was diagnosed due to symptoms of fever, rash erythema, effusion, and granuloma formation without evidence of infection [22]. In 2014, the same group presented another retrospective review on metal allergy after MIRPE in which the incidence increased to 6.4% [7]. The authors stated that the clinical significance of an allergic reaction should not be underestimated [7]. One patient even developed a metal allergy after MIRPE despite a preoperative negative skin test [9].

First line therapy of the allergic symptoms includes steroids and antibiotics. In many cases, the symptoms resolve with time. In severe cases, in which the patient fails to respond to steroids or unacceptable high doses of steroids are needed, the metal bar has to be prematurely explanted [10, 22]. We also experienced the high incidence of reactions to the metal bar and postulate that a high release of metal ions from the implants after MIRPE could be the cause of this.

Until now, the contamination with metal debris following MIRPE has not been studied in depth. One Australian study reported levels of chromium, molybdenum, and nickel in blood in a series of 11 patients who had undergone the Nuss procedure. The researchers collected the blood samples at different time points after the procedure at a mean time of 13 months (range 4–21 months) and compared the level of metal in these blood samples with those of patients without metal implants. They described significantly elevated chromium levels in patients after MIRPE. Nickel levels were also elevated after MIRPE; however, due to high variability and the low numbers of patients involved in the study, these differences were not statistically significant [23].

Detection of metal contamination is complex and has many variables. Metal debris can accumulate in various compartments and at different time points. Thus, the analysis is difficult, and it is recommended to evaluate contamination by repeated measurements in different compartments at different time points. Nonetheless, the observed increase in metal ion concentration is in line with our hypothesis.

To study this situation in depth, we assessed the local tissue as well as systemic metal contamination by examining the concentrations of metal in the tissue surrounding the bar and, in addition, analyzed the concentrations in blood and urine. To reduce variability, we compared patients prior to MIRPE with patients who underwent explantation of the bar after three years; i.e., at very defined time points.

Our study found that patients who had the bar(s) in place for a common treatment period of three years exhibited significantly higher mean values of chromium in urine and nickel in blood than the control group. However, it was difficult to determine the biologic relevance of the detected elevated metal values. Like contact dermatitis, every patients’ reaction to the metal is highly individual. A large number of individuals, even nickel-sensitized patients, will tolerate low levels of metals in implants without developing a reaction [8].

To improve the expressiveness of our detected metal values, we used reference values as a guideline. The reference values function as an orientation guide and suggest only intoxication if clinical symptoms occur simultaneously to elevated metal values. Thus, the presence of trace metal contamination in combination with the clinical symptoms observed after MIRPE in this study and others suggests trace metal toxicity as the cause of these symptoms. The chromium contamination, which has a carcinogenic compound, is also alarming, especially because of the fact that the patients involved are typically adolescents [24]. The urinary excretion of chromium is not fully effective and this metal tends to accumulate in the tissue and the red blood cells [25]. The mean levels of chromium in urine and nickel in blood detected in the current study were also above the reference values. These results indicate that patients who underwent MIRPE receive an additional exposure to trace metal ions.

Numerous studies that have assessed patients who have undergone total hip arthroplasty have reported an elevation of cobalt and chromium in combination with local adverse reactions and an increased risk of systemic accumulation of trace metal emissions [15]. The local toxicity is caused by the response of macrophages and probably some immunological reaction involving hypersensitivity and results in aseptic loosening and osteolysis [24]. Systemically metal ions can have numerous effects; e.g., a wide spectrum of immunomodulatory effects by interacting with T- and B-lymphocytes after being phagocytosed by macrophages [24]. The comparison between these studies and our study is difficult because the surgical steel used in MIRPE has a different composition to the Co-Cr alloys that are used for hip arthroplasty. Studies that have focused on cobalt and chromium have found that wear and passive corrosion are both factors that increase ion concentration in body fluids [26]. In MIRPE, we use stainless steel, which is known to have a lower corrosion resistance and susceptibility to wear; as such, there is a higher risk that potentially damaging metal ions will be released [27, 28]. Another difference is that the metal is implanted into soft tissue.

The underlying cause of the elevation of nickel and chromium after implantation of the stainless steel bar could be metal debris caused by wear, especially between the bar and metal stabilizers. Our macroscopically observation of signs of wear on the contact surfaces of some explanted bars and stabilizers would support this hypothesis. Another explanation could be a diffuse release of metal ions from the surface of the bar, especially in the areas that come in contact with the pleura, which is an immunologically very active compartment with many macrophages that could trigger foreign body responses [29]. It is also considered that bending the bars leads to changes of the surface and causes little fractures resulting in metal release. We hypothesize that the trace metal contamination causes the observed allergic symptoms.

There are several drawbacks of the study. The number of patients is small and a power analysis could not be performed based on the lacking data in the literature. In addition, we did not investigate the same patient group before bar implantation and at bar removal. Further studies are needed to amplify this research.

The underlying mechanism of the high contamination with trace metal ions in patients undergoing pectus repair remains to be determined. Although our study sought to detect the highly variable metal contamination [30] at a single time point in a limited number of patients, our data suggests that trace metal contamination is a relevant problem in MIRPE patients. This could potentially lead to the high incidence of allergic symptoms in these patients. Thus, the producers of medical implants should work on improving designs and materials to address it.
